# Clinical Status, Nutritional Behavior, and Lifestyle, and Determinants of Community Well-Being of Patients from the Perspective of Physicians: A Cross-Sectional Study of Young Older Adults, Nonagenarians, and Centenarians in Salerno and Province, Italy

**DOI:** 10.3390/nu14173665

**Published:** 2022-09-05

**Authors:** Silvana Mirella Aliberti, Richard H. W. Funk, Luigi Schiavo, Aldo Giudice, Elena Ciaglia, Annibale Alessandro Puca, Joseph Gonnella, Mario Capunzo

**Affiliations:** 1 Department of Medicine, Surgery and Dentistry “Scuola Medica Salernitana”, University of Salerno, 84081 Salerno, Italy; 2 Medical Faculty Carl Gustav Carus, Institute of Anatomy, University of Technology (TU) Dresden, 01307 Dresden, Germany; 3 Animal Facility, Istituto Nazionale Tumori—Istituto di Ricovero e Cura a Carattere Scientifico (IRCCS)—“Fond. G. Pascale”, 80131 Naples, Italy; 4 Cardiovascular Research Unit, IRCCS MultiMedica, 20138 Milan, Italy; 5 Center for Medical Research in Medical Education and Health Care, Sidney Kimmel Medical College, Thomas Jefferson University, Philadelphia, PA 19107, USA; 6 Complex Operational Unit Health Hygiene, University Hospital “San Giovanni di Dio e Ruggi d’Aragona”, 84131 Salerno, Italy

**Keywords:** old population, centenarians, clinical status, nutritional behavior, lifestyle, community well-being, healthy living, physicians

## Abstract

Longevity is rightly considered one of the greatest achievements of modern society. Biomedical research has shown that aging is the major risk factor for many diseases, so to find the right answers to aging it is necessary to identify factors that can positively influence longevity. This study investigated the clinical status, nutritional behavior, lifestyle, and social and community determinants of the well-being of young older adults and nonagenarians/centenarians in Salerno and province through the judgment of their physicians. Data were collected through an online survey. Multivariate Poisson and logistic regression models were used to calculate significant predictors of the outcomes of interest. The interesting finding was that cardiovascular disease was a risk factor for young older adults, while it was a protective factor for nonagenarians/centenarians, meaning that as age increased, heart problems tended to decrease. Certain foods were found to be a significant protective factor for both young older adult and nonagenarian–centenarian patients. In addition, psychosomatic disorders were found to be determinant for the young older adults, while depression was a risk factor for the nonagenarians/centenarians because they were not always gratified by their long lives and often felt like a burden on the family. The protective significant variable among the determinants of community well-being for both young older adults and nonagenarians/centenarians was the retention of honorary achievement. Based on our results, we are able to support the hypothesis of a difference between the young older adults and the nonagenarians/centenarians in clinical status, nutritional behaviors, lifestyle, and determinants of community well-being. However, societies need more social and educational programs that are able to build “a new idea of old age” by improving and supporting the young older adults and the nonagenarians/centenarians, with the goal of intergenerational solidarity, well-being, and social inclusion, as well as preventive interventions on lifestyles and nutrition, which will allow us to provide a new key to understanding aging.

## 1. Introduction

The world is home to 727 million older people (65 years and older) and half-a-million centenarians (100 years and older) worldwide [1], and this trend will tend to increase in the future as birth rates decline and life expectancy increases. Seen in this way, the aging process is indeed a major social concern, as one wonders how a population composed mainly of older people can meet the needs of economic development and growth, as well as welfare, care, and social assistance.

The aging process can be viewed with a more nuanced view of the phenomenon than the worried image. In fact, longevity is rightly regarded as one of the greatest achievements of modern society, an achievement understood as the possibility of increasing the healthy part of life and not only its duration. In this regard, territorial medical doctors (DTMs) play an important role in monitoring the health status of the population, with particular reference to the old and centenarians, who are more prone to age-related diseases and thus the need to adjust drug therapies over time. This monitoring makes it possible to intercept the onset of potential diseases and/or their aggravation and consequently decrease the increase in expenditure for the National Health System (NHS) in terms of direct and indirect costs. Therefore, physicians represent specialists (stakeholders) who can provide a careful assessment of the health, diseases, and problems that afflict the old and centenarian population. On the other hand, from a physiological point of view, the old and the centenarians constitute a heterogeneous population, with individuals who retain relatively good physical and functional conditions and others with severe pathologies and consequent reduction of autonomy [2]. The interindividual differences can be summarized by dividing the old into three groups: a first substantial group consists of individuals who reach very advanced ages without presenting any apparent pathology, others become ill only in the very last years of life, and a third group consists of those who could survive for long years despite the presence of chronic diseases Therefore, the identification of factors that can protect the old population from becoming ill is undoubtedly of great importance in health management [3], although Vaupel et al. [4] stated that there is a deceleration in mortality rates after the age of 80, a phenomenon that has been termed “morbidity compression” by Fries [5]. The cause of the deceleration in mortality rates is due to the decrease in population heterogeneity in old age [6] or the selection of a surviving population carrying protective genetic factors and lacking risk factors for killer diseases [7].

According to some authors [8,9,10] an important role in the mechanisms that regulate the attainment of advanced age could be played by genetic polymorphisms that regulate the immune response. Homocysteine concentration [11,12] would seem to be an additional factor in close relation to longevity. Its increase has been associated with ischemic cardiac events, stroke, venous thrombosis, Alzheimer’s disease, osteoporosis, and depression [13,14,15], although the results of studies are often discordant [16]. Other studies would indicate an important role of p53, a protein that would fill the function of a tumor suppressor at a young age, which has also been recently associated with the regulation of aging [17,18,19,20]. Vecchione et al., added an additional study conducted in patient groups showing that a higher level of BPIFB4 protein in the blood corresponds to better blood vessel health. This is a protein isolated years ago in long-lived individuals over 100 years old. Subjects with the highest levels of this particular protein were also “immune” to stroke or cardiovascular disease. The researchers’ goal is to be able to transfer the genetic advantages of the long-lived people to the general population, so that even people who do not possess those particular genetic characteristics that make them long-lived can be offered the same level of protection [21,22].

Several studies have found that human longevity is an estimated 15 to 40% heritable component [23,24,25,26,27,28,29], while Ruby et al. [30] pointed out, due to assorted mating, the true genetic component of longevity, widely overestimated in the past, is probably less than 10%.

Among the myriad of genetic factors, it is important to understand which factors are able to modify the epigenome to establish healthy aging. Epigenetics [31,32,33] may provide a particular answer to this question. In humans, the factors that may influence epigenetic status and may be associated with health status and longevity can be divided into: environment, nutrition, medical care, and lifestyle.

In agreement with Aliberti et al. [34], environmental factors such as hill altitude, transitional climate between the Mediterranean and the temperate climate, UNESCO heritage areas, and hinterland areas can directly or indirectly influence local longevity in Cilento and centenarians.

According to Darviri et al. [35] longevity has also been associated with specific lifestyles, such as the tendency to avoid conflict situations and the ability to respond positively to stress. In a survey of the rural population of Cilento, Scelzo et al. [36] found that study participants over the age of 90 had better mental well-being than younger ones, due to their resilience and optimism and attachment to family and religion. Pizza et al. [37], on the other hand, emphasized the importance of lifestyle, nutrition, and individual personality factors. According to Franceschi et al. [38], improved socio-ecological conditions, medical care, and quality of life have been identified as factors for an overall improvement in the health status of the population. Healthy nutrition and moderate physical activity are also accepted as key components in the prevention of processes that promote aging [39,40].

Despite numerous studies on the topic of aging, to the best of our knowledge, no research to date has analyzed health or disease status, behaviors, nutrition, lifestyle, or social and community determinants from the perspective of physicians.

The objectives of the study were to assess the clinical status, respectively, of the groups of young older adults and nonagenarians/centenarians; to evaluate their nutritional and lifestyle behavior; to understand the social and community determinants related to their well-being, in the Salerno area and province, through the expert judgment of their physicians.

## 2. Materials and Methods

### 2.1. Study Design and Sampling

The study is a cross-sectional survey conducted between December and June 2020–2021 among a random sample of physicians in Salerno and province to assess the health or disease status of young older adults and nonagenarians/centenarians, respectively, and to understand which factors such as nutrition, behaviors, mental health, and social and community gradients are positively or negatively correlated with measures of well-being and healthy living and may contribute to improved quality of life and possibly local longevity.

**Figure 1:**
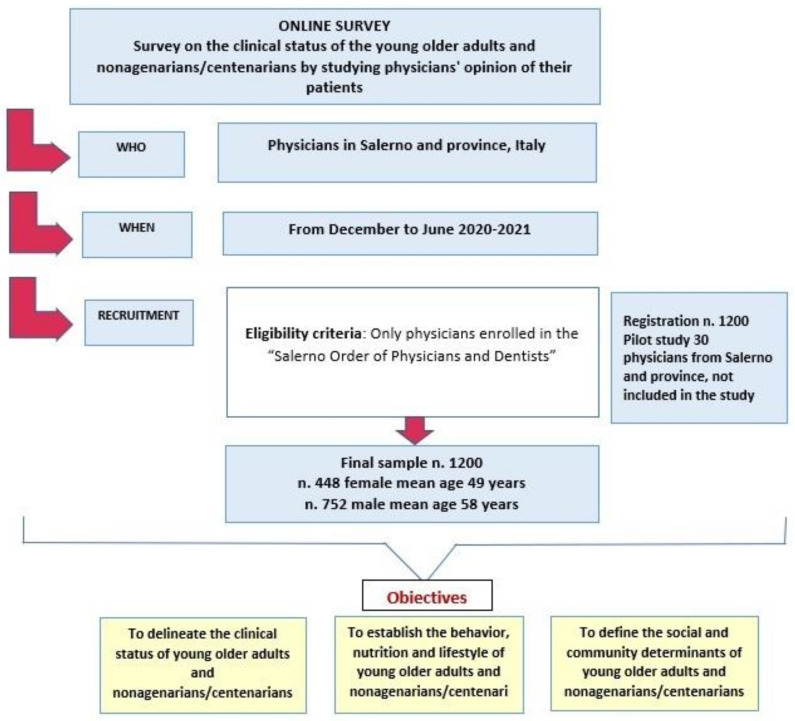
Survey design, including data collection, participant recruitment, and objectives. Detailed information can be found in the introduction, study design, and results section.

Eligibility criteria included only physicians enrolled in the “Salerno Order of Physicians and Dentists” (Figure 1).

The sample size was calculated with the following equation [41]:

(1) $$n=\frac{Z^{2}\;P\left(1-P\right)}{d^{2}}\;$$

where *n* is the sample size, *Z* is the *Z* statistic related confidence level, P is the expected prevalence or proportion, and d is the precision. In our study the *Z* value is 1.96 for a 95% confidence level, the prevalence is 85% (in proportion of one *P* is equal to 0.85) of physicians who responded to the survey, the level of precision is 2% (in proportion of one *d* = 0.02), and the sample size recommended was 1063.

### 2.2. Data Collection Procedure

Research participants were contacted with the support of the “Salerno Order of Physicians and Dentists”, and were provided explanations of the purpose and methods of the study and asked physicians to collaborate in the survey. Physicians were invited to participate in the research with a letter published on the web pages and social networks of the same order. The physicians, in turn, obtained the information on the health status of their patients from their updated database, while nutritional behavior and lifestyle were obtained from the physicians themselves through the administration of questionnaires.

Data on the administration of questionnaires to physicians were collected via LimeSurvey (Hamburg, Germany), a professional online survey platform, which provides: (1) an intuitive interface for data entry; (2) audit trails to monitor data manipulation and export procedures; (3) automated export procedures for downloading data into common statistical packages; (4) procedures for importing data from external sources [42]. The online survey was anonymous and self-reported; the only sociodemographic items required were gender, age, and professional competence. An implicit statement of consent was obtained from the participants, as the questionnaire was administered via an electronic tool (each questionnaire was completed once based on IP address), which physicians were specifically and intentionally required to access on the internet via a link (https://old-people.limequery.com/243344?lang=it, accessed on 15 July 2021). However, in the header of the webpage questionnaire, to exclude any liability, a text explained the objective of the study and the anonymous and voluntary nature of participation.

### 2.3. Data Collection Instrument

Between July and October 2020, three focus groups were held with stakeholders on the topic of an aging population and the transformations this entails in various areas of society, from the work environment to the healthcare system and from the economy to family structures and intergenerational ties. The content analysis of the focus groups was decisive in outlining the content of the questionnaire. The questionnaire was constructed based on the information collected and then shared with the research topic experts, who reviewed all the questionnaire items for readability, clarity, and completeness [43]. The questionnaire was then pretested with a random sample of 30 physicians from Salerno and province (Figure 1). After the pretest, some modifications were made to further improve the readability, clarity, and comprehensibility of the questionnaire and after establishing a level of agreement the final version was approved by the team of raters. The results of the pretest were not included in the study.

Content validity was supported by factor analysis of the different models, and reliability was measured by the α coefficient, which ranged from 0.79 to 0.87 in the models considered.

The questionnaire was written in Italian and German and then also translated into English (Supplementary Material S1).

The instrument consisted of six main sections, divided according to the model of Dahlgren and Whitehead [44]: (1) sociodemographic characteristics of surveyed physicians (gender, age, occupation, and education level of the respondent); (2) physician’s assessment of the clinical status of the young older adults and nonagenarians/centenarians (age groups divided into 65–89 as young older adults (aging tendency and 85+ ratio) and 90–115 as nonagenarians/centenarians) [34]; estimated health or disease of patients in the past three years, definition of most common diseases, estimated comorbidities, amount of patients infected with SARS-CoV-2. Questions included “completely healthy”, “little limited ability”, “marked by disease”, or “suffers a lot”, a horizontal analog scale (rating scales) to assess the amount of young older adults and nonagenarians/centenarian patients, a horizontal analog scale (rating scales) that took values between 0 (best condition) and 100 (worst condition), open-ended “yes” or “no” and multiple-choice questions; (3) physician’s assessment of the behavioral, lifestyle, and nutritional factors of the young older adults and the nonagenarians/centenarians (use of drugs, tobacco and alcohol, nutrition, mental health). Six-point Likert scales were used for responses, with endpoints labeled as 1 = especially high and 6 = extremely low; seven-point Likert scales, with endpoints labeled as 1 = very relevant and 7 = completely irrelevant; horizontal analog scales (rating scales) that took values between 0 (best condition) and 100 (worst condition); responses included “yes”, “no”, and “undecided”; (4) physician’s assessment of social and community determinants of the young older adults and nonagenarians/centenarians (family, caregiver, social support, professional positions, honorary achievement, etc.), horizontal analog scales (rating scales) that took values between 0 (least support) and 100 (most support), and open-ended questions; (5) physician’s assessment of structural determinants (evaluation of the health system organization for equipment, collaboration, information). Six-point Likert scales with endpoints labeled as 1 = very good and 6 = extremely poor and open-ended questions were used for responses.

### 2.4. Statistical Analysis

Descriptive statistics were used to summarize participant characteristics, and responses to all items were shown with absolute and relative frequencies for categorical variables and mean and standard deviation for continuous variables. Univariate analyses were performed using chi-square test for categorical variables and Student’s t-tests for continuous variables, as appropriate. Next, variables with a *p*-value less than or equal to 0.25 were included in the multivariate Poisson and logistic regression models, and the significant level choices for inclusion and elimination of variables in the models were *p*-values of 0.2 and 0.4, respectively, in agreement with Hosmer and Lemeshow [45].

Multivariate analysis was used to identify significant predictors of the following outcomes: the young older adults (65–89 years) and the nonagenarians/centenarians (90–115 years), respectively (continuous) (Model 1–3, 5); importance of nutrition in the young older adults and the nonagenarians/centenarians, respectively, which was dichotomized into “very high” = 0 versus “high” = 1 (rather high, so-so); questions regarding “rather unimportant”, “extremely low”, and “don’t know” were not considered, because there were zero responses (Model 4); gender of physicians (male = 0, female = 1) (Model 6).

The following selected independent variables were included in the models: completely healthy, little limited ability, clearly marked by diseases, suffer a lot (score <50 = 0; score ≥50 = 1), cardiovascular diseases, respiratory diseases, blood diseases, rheumatic diseases, metabolic diseases, gastrointestinal diseases, neurological diseases, biliary diseases, oncological diseases, other diseases (preference responses from 1 to 10 were recoded as 1–5 = 1; 6–10 = 0), comorbidities (continuous) (Model 1); asymptomatic, paucisymptomatic, symptomatic, symptomatic with severe diseases, dead (continuous) (Model 2); use of drugs, tobacco, alcohol, addiction, smoking addiction, psychosomatic problems, depression, respectively (continuous), nonagenarians/centenarians grateful for a long life and suffering the burden of not dying were measured in undecided = 1 (baseline result), no = 2, yes = 3 (Model 3); variety and freshness of foods, consumption of ready-to-eat meals, bottled water, tap water, meat consumption, fish consumption, specific diets (all responses were dichotomized into very important, important, rather important = 0; rather unimportant, unimportant, totally unimportant, do not know = 1), overweight (continuous) (Model 4); living with a family member, living alone with family support, living alone with third-party support (neighbors, caregivers), living in nursing homes or shared apartments (continuous), continuing in professional positions, honorary achievement, physical activities (gardening, yoga, sports, etc.), cultural activities, social life, parish activities, bar meetings, association activities, all responses were dichotomized into important = 0 (very important, important, rather important) versus unimportant = 1 (rather unimportant, unimportant, totally unimportant, do not know) (Model 5); hospital equipment, cooperation between institutions, public health information, cooperation between clinics and primary care physicians, cooperation between clinics and rehabilitation facilities, cooperation between hospitals and specialty clinics, self-help group (all responses were recoded to very good, good = 0; so-so = 1; poor, extremely poor = 1) (Model 6).

To examine the contribution of each variable, incidence rate ratios (IRRs) and associated confidence intervals (CIs) were calculated in the multivariate Poisson regression model and odds ratios (ORs) and associated confidence intervals (CIs) were calculated in the multivariate logistic regression analysis. All significance tests were two-sided, and *p* values equal to or less than 0.05 were considered statistically significant. Data analyses were conducted with STATA [46].

## 3. Results

### 3.1. Sociodemographic Data of Physicians Who Participated in the Survey

The sample consisted of 1200 physicians who agreed to be interviewed. Nearly three-fifths of the sample were male, with an age of 58 (±11.9) years; the remaining two-fifths were female, with an age of 49 (±12.9) years. The largest respondents were general practitioners, geriatricians, surgeons, internists, cardiologists, and dentists (Supplementary Material S2).

### 3.2. Physician’s Assessment of the Clinical Status of the Young Older Adults and the Nonagenarians/Centenarians

Physicians who participated in the survey reported that 63.6 ± 25.1 of their patients were young older adults (65–89 years old) and 10.6 ± 13.4 were nonagenarians/centenarians (90–115 years old). Regarding clinical status, it was observed that healthy young older adults were 21.6 ± 15.1, those with little limited capacity 38 ± 23, and those with higher morbidity 40.4 ± 24.4. The diseases with the highest frequency among young older adult patients were cardiovascular, metabolic, respiratory, neurological, rheumatic, and oncological diseases. The analysis of data for nonagenarians/centenarians showed that 13.7 ± 16.6 were healthy, 25.2 ± 22.3 had little limited ability, and 57.9± 44.9 had higher morbidity. For nonagenarians/centenarians, cardiovascular diseases remained in first place, but the ranking tended to change, followed by metabolic, respiratory, neurological, gastrointestinal, and blood diseases. Based on the previous analysis of the age and clinical evidence of the diseases, the nonagenarians/centenarians fell into three categories, respectively: escapers (had an age of onset of 100 years or had not yet been diagnosed with a disease), survivors (had an age of onset of less than 80 years for at least one of the diseases), and delayers (had an age of onset between the age of 80 and 100 years) [47].

The prevalence of comorbidities, as assessed by surveyed physicians, was 62.2 ± 21.6 for the young older adults and 52.6 ± 33.5 for the nonagenarians/centenarians. Table 1 (Model 1) presents the results of the multivariate Poisson regression models constructed to investigate the role played by different explanatory variables in the outcomes of interest. The multivariate Poisson model constructed to study clinical status showed that for the young older adults, the variables “suffer a lot”, respiratory, rheumatic, gastrointestinal, neurological, and, oncological diseases, had a positive association, meaning they represent risk factors; for the nonagenarians/centenarians, the variables “little limited ability”, “clearly marked by diseases”, “suffer a lot”, and most of the diseases listed in the study, had a positive association (i.e., represent risk factors). Cardiovascular diseases were a risk for the young older adults while it was a protective factor for the nonagenarians/centenarians, meaning that as age increases, heart problems tend to decrease. Being “completely healthy” was a protective factor for the nonagenarians/centenarians, while it was a risk factor for the young older adults.

With regard to SARS-CoV-2 infections, responding physicians reported that one third of patients, young older adults and nonagenarians/centenarians, were asymptomatic (mean 27.5 and 28.1), and a mean 5, respectively, died. Multivariate analysis showed several associations with young older adult and nonagenarian/centenarian outcomes (Model 2 in Table 1).

### 3.3. Physician’s Assessment of Behavioral, Lifestyle, and Nutritional Factors of the Young Older Adults and the Nonagenarians/Centenarians

Regarding behaviors and lifestyle, the young older adults were more likely to use tobacco (28.2 ± 16.9) and alcohol (26.6 ± 20.3) than the nonagenarians/centenarians (6.9 ± 11.8 and 11.3 ± 15, respectively), but they did not represent addiction. A total of 43.8 ± 25.8 of the young older adults suffered from psychosomatic complaints, such as symptoms of insomnia, constipation, and fatigue, which was a significant determinant. Depression was a risk factor for the nonagenarians/centenarians, probably because they were not always gratified by their long lives and they often felt like a burden on the family (Model 3 in Table 2).

**Figure 2:**
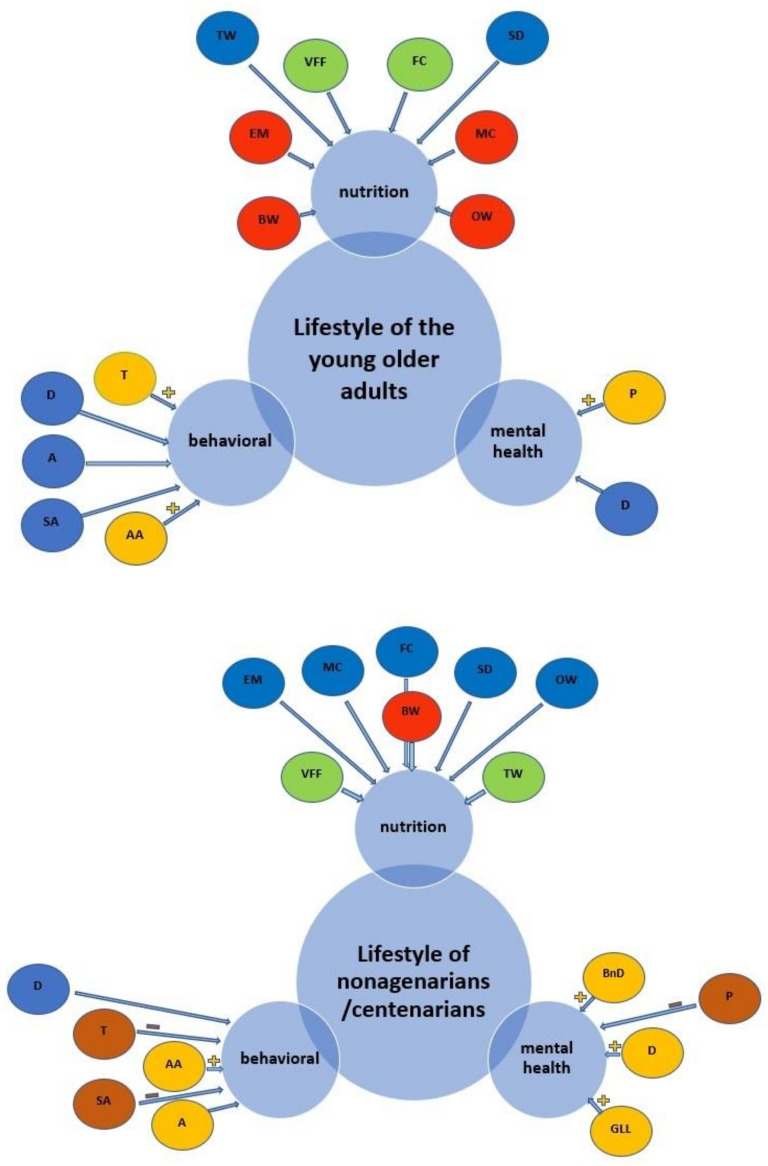
Structural patterns of young older adults’ and nonagenarians/centenarian’ associations with lifestyle. 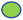 protective factors that are statistically significant; 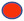 risk factors that are statistically significant; 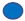 factors that are not statistically significant; 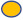 positively correlated factors; 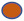 negatively correlated factors. **VFF**—variety and freshness of foods; **EM**—consumption of ready-to-eat meals; **BW**—bottled water; **TW**—tap water; **MC**—meat consumption; **FC**—fish consumption; **SD**—specific diets; **OW**—overweight; **D**—use of drugs; **T**—tobacco; **A**—alcohol; **AA**—alcohol addiction; **SA**—smoking addiction; **P**—psychosomatic problems; **D**—depression; **GLL**—centenarians grateful for a long life; **BnD**—centenarians suffered the burden of not dying. For more information look at Table 2.

Nutrition was a significant factor in patient care; in fact, 752 (64.8%) physicians believed that it had a “very significant” influence on the health of the young older adults and the nonagenarians/centenarians. Logistic regression showed that the nutritional aspects that had the greatest protective value for the well-being of both the young older adults and the nonagenarians/centenarians were the “variety and freshness of foods” (OR = 0.08, *p* <0.001; OR = 0.35, *p* <0.001). For the nonagenarians/centenarians, tap water consumption was also found to be a protective factor (OR = 0.40, *p* <0.001). For the young older adults, in particular, the use of convenience foods and a meat-rich nutrition were found to be a risk factor (Figure 2). Being overweight was also found to be a risk factor for longevity (Models 4 in Table 2).

### 3.4. Physician’s Assessment of the Social and Community Determinants of the Young Older Adults and the Nonagenarians/Centenarians

Physicians’ responses indicated that 34.4 ± 29.7 of the young older adults and 36 ± 31.9 of the nonagenarians/centenarians received family support; 27.4 ± 23 of the young older adults and 18.2 ± 17.8 of the nonagenarians/centenarians lived alone with family support; 27.8 ± 20.7 of the young older adults and 25.1 ± 21.1 of the nonagenarians/centenarians lived alone but were assisted by third persons (caregivers, social workers, neighbors); finally, 21.2 ± 17 of the young older adults and 27.2 ± 20.1 of the nonagenarians/centenarians lived in nursing homes or shared apartments. It was important for the young older adults and the nonagenarians/centenarians to keep themselves engaged in various activities. The multivariate Poisson regression model showed that for nonagenarians/centenarians a significant protective factor may be “living with family member” and “living alone with third-party support”. As for the significant protective variables of community determinants for both young older adults and nonagenarians/centenarians, they were: continuation of professional activities (IRR = 0.93, *p* = 0.042; IRR = 0.59, *p* <0.001), retention of honorary achievement (IRR = 0.93; *p* <0.001; IRR = 0.75, *p* <0.001), and participation in cultural activities (IRR = 0.66, *p* <0.001; IRR = 0.59, *p* <0.001) (Model 5 in Table 3).

### 3.5. Physician’s Assessment of the Organization of the Health Care System

The Salerno Health System was evaluated by the physicians who participated in the survey, taking into account the gender difference (males 752 (62.7%), females 448 (37.3%)). From the data analysis, it was observed that both male (MM) and female (FM) physicians rated the availability of “hospital equipment” as fair; 372 out of 752 (51%) of the MMs versus 160 out of 448 (36.3%) of the FMs rated “cooperations between institutions” as inadequate; 352 out of 752 (47.8%) of the MMs and 176 out of 448 (40%) of the FMs rated public health information as fair. As for cooperation between clinics and primary care physicians, it was rated as poor by 288 out of 752 (39.1%) of the MMs versus 224 out of 448 (50.9%) of the FMs. Cooperation between clinics and rehabilitation facilities, between hospitals and specialized clinics and self-help groups was rated as fair by both genders. The results of the multivariate logistic regression model revealed that statistically significant predictors in physicians’ evaluations of the health care system included poor hospital equipment and poor collaboration between clinics, primary care physicians, and rehabilitation facilities (Model 6 in Table 4).

## 4. Discussion

The main objectives of this study were to investigate the clinical status of young older adults and nonagenarians/centenarians, to determine their nutritional and lifestyle behavior, and to understand which social and community determinants are related to the predictive factors considered. The report on the evaluation of the Salerno province health care system and their regressors were also presented. The survey was aimed at physicians, as they represent the specialists (stakeholders) who can provide a careful assessment of the health, diseases, and problems that afflict the young older adults and the nonagenarians/centenarians.

The survey conducted with physicians in Salerno and province showed that the nonagenarians/centenarians did not have the same health status compared with the young older adults. The interesting finding was that cardiovascular diseases were a risk for young older adults while they are a protective factor for nonagenarians/centenarians, meaning that as age increases, heart problems tended to decrease. The explanation may come from the studies by Puca et al. [22] on the so-called “longevity gene”, LAV-BPIFB4 (longevity-associated variant), a variant of the gene encoding the BPIFB4 protein found in the DNA of people over 100 years old. In experimental studies, researchers observed that administration of the BPIFB4 protein encoded by the LAV-BPIFB4 gene to the human blood vessels of patients with atherosclerosis resulted in improved vascular activity, reduced blood pressure, and increased resistance to cellular stress. The result was a true rejuvenation of blood vessels and the cardiovascular system. This means that centenarians have genetic protection against cardiovascular disease.

According to the physicians surveyed, the most worrying finding was the presence of multimorbidity, which can lead to complications, the possibility of increased hospitalization, and poor quality of life for young older adult and nonagenarian–centenarian patients. Our data are in agreement with the research of von Berenberg et al. [48], who compared the proportion of centenarians who have chronic conditions and who use health care services in different care setting. In this context, Abete et al. [49] showed that the combination of chronic diseases in the old patient, such as organic heart disease and osteoporosis, increases the relative risk of disability and consequently decreases quality of life.

A second interesting aspect that emerged from the survey concerns the nutritional and lifestyle behavior of young older adults and nonagenarians/centenarians. Nutrition was a significant factor for both the young older adults and the nonagenarians/centenarians. For nonagenarians/centenarians, variety and freshness of foods and consumption of tap water were found to be protective factors. This is in agreement with our previous study [50], which emphasized that the consumption of plant foods, olive oil, and some wine with meals was beneficial to health. This should not be surprising, considering that numerous studies have shown that nutrition is one of the most important components for prolonging healthy life [51,52,53,54,55,56] and that, in order to slow aging and counteract the onset of major age-related diseases, it is necessary to follow a healthy diet from a young age [57]. In addition to nutrition, of key importance for health and longevity may also be the trace elements in water [58,59,60]; in fact, as our research results on nonagenarians/centenarians found, tap water may also have protective effects.

One aspect not to be overlooked on the behaviors and lifestyle of the young older adults and the nonagenarians/centenarians, according to the results of our research, were the psychosomatic problems that affect the young older adults. Sadness, loss of health, feeling worried about even trivial matters, and insomnia are psychodynamic factors that can affect the young older adults and can result in depression in the nonagenarians/centenarians [61]. Indeed, our research showed that a worrying finding may be depression in the nonagenarians/centenarians, who were not always gratified by their long life and sometimes felt like a burden on the family.

A third interesting aspect that emerged from the survey concerns the support nonagenarians/centenarians receive from family or third parties. In the past, unpaid female labor has traditionally been the most important source of support for the dependent old, while today, with the increased inclusion of women in the workforce, there is a progressive reliance on third-party caregivers [62]. According to survey data, the figure of the “caregiver” is increasingly being used. In addition, with advancing age, it is necessary to adopt behaviors and attitudes that are more attentive to one’s health, balance, and mental and physical well-being. As noted in the survey, aging well requires initiatives implemented by individuals and the community, such as cultural activities or honorary achievement, which can have a significant implication at the level of personal well-being.

Uncomforting data emerge from the fourth aspect analyzed, which concerns physicians’ opinions of the Salerno Health System, considering their professional experience. Physicians believe that a change of course is needed in health care, with a new operational structure that favors new ways of producing services and greater collaboration between the territory, hospitals, and physicians. Karagiannis et al. [63] also stated that a team-based culture of care needs to be created so that the high prevalence of chronic diseases in the old population can be managed. Since hospitals have to deal mainly with the care of serious cases, there is no doubt that home care should be improved. Territorial medicine could play a decisive role in avoiding unnecessary hospitalizations. Morselli et al. [64] argued that it is necessary to rethink the structural structure of the health service, with the aim of making it more effective and efficient.

According to the physicians who responded to the survey, the organizational structure of health care had to provide for improved quality of services, better accessibility of services by users, and a more human and welcoming dimension by health care staff. This was in agreement with Hojat et al. [65], who reported that empathic engagement is the basis of a trusting relationship. This in turn leads to more accurate diagnoses and greater compliance, which ultimately results in better quality care [65].

To the best of our knowledge, this is the first study in which the determinants of clinical, nutritional, behavioral, and lifestyle status as well as social and community well-being of young older adults and nonagenarian–centenarian patients were assessed from the perspective of physicians. However, the present study has, in our opinion, the following limitation. We conducted our research only on a specific group of physicians from the province of Salerno; therefore, a multicenter study with a larger number of physicians from different Italian regions or even a comparative study with other nations may be needed to confirm our data. In addition, our survey was based solely on self-reported data from physicians and implicit biases are inevitable with subjective data.

## 5. Conclusions

In summary, the results of this survey, obtained by analyzing data provided by surveyed physicians, suggest that about three-fifths of young older adults and two-fifths of nonagenarians/centenarians, respectively, enjoy good health status in Salerno and province, Italy. The greatest risk is multimorbidity, which could afflict both young older adults and nonagenarians/centenarians and can lead to the demand for health care. Among the different diseases analyzed to define clinical status, it was observed that heart problems tended to decrease with increasing age, meaning that cardiovascular disease was a risk factor for young older adults, while it was a protective factor for nonagenarians/centenarians. However, a major problem for nonagenarians/centenarians was that they were not grateful for their long lives, as some felt they were a burden on the family. Significant protective factors were found to be certain foods for both young older adults and nonagenarians/centenarians, while tap water only for nonagenarians/centenarians. Factors that determined community well-being included the retention of honorary achievements and cultural activities.

Regarding health care in Salerno and province, Italy, considering the complex relationship between longevity and healthy aging, further steps are needed to optimize future services and better meet patients’ needs.

Societies need more social and educational programs that are able to build “a new idea of old age” by improving and supporting the young older adults and the nonagenarians/centenarians, with the goal of intergenerational solidarity, well-being, and social inclusion, as well as preventive interventions on lifestyles and nutrition, which will allow us to provide a new key to understanding aging.

## Figures and Tables

**Table 1:** Clinical status, respectively, of the younger older adults and the nonagenarians/centenarians.

**Model 1**	**Younger Older Adults (Outcome)** **Log Likelihood= −8822.6,** **x^2^ = 2873.08 (15 df), *p* < 0.001**			**Nonagenarians/Centenarians (Outcome)** **Log Likelihood= −7728.0,** **x^2^ = 3632.16 (16 df), *p* < 0.001**		
Clinical Status Variables *	**IRR**	**95% CI**	***p***	**IRR**	**95% CI**	***p***
Completely healthy	1.28	1.24–1.31	<0.001	0.90	0.84–0.97	0.009
Little limited ability	0.98	0.96–0.99	0.024	1.31	1.25–1.37	<0.001
Clearly marked by diseases	0.96	0.94–0.98	<0.001	1.22	1.17–1.27	<0.001
Suffer a lot	1.11	1.08–1.14	<0.001	1.42	1.36–1.48	<0.001
Cardiovascular diseases	1.04	1.02–1.06	<0.001	0.82	0.77–0.86	<0.001
Respiratory diseases	1.11	1.09–1.13	<0.001	1.24	1.19–1.29	<0.001
Blood diseases	0.99	0.97–1.01	0.634	1.40	1.34–1.45	<0.001
Rheumatic diseases	1.06	1.05 - 1.08	<0.001	1.20	1.15–1.25	<0.001
Metabolic diseases	0.93	0.91–0.95	<0.001	1.15	1.10–1.19	<0.001
Gastrointestinal diseases	1.14	1.12–1.16	<0.001	1.23	1.18–1.29	<0.001
Neurological diseases	1.11	1.09–1.13	<0.001	1.87	1.77–1.98	<0.001
Biliary diseases	0.89	0.87 - 0.92	<0.001	0.44	0.41–0.48	<0.001
Oncological diseases	1.01	0.99–1.02	0.136	1.09	1.03–1.14	0.001
Other diseases	1.20	1.17–1.23	<0.001	1.76	1.67–1.86	<0.001
Comorbidities	1.00	1.00–1.00	<0.001	1.00	1.00–1.00	<0.001
**Model 2**	**Log Likelihood = −3212.6,** **x^2^ = 611.68 (5 df), *p* < 0.001**			**Log Likelihood = −2212.1,** **x^2^ = 396.84 (6 df), *p* < 0.001**		
Asymptomatic	1.00	1.00–1.00	<0.001	0.99	0.99–1.00	0.286
Paucisymptomatic	0.99	0.99–0.99	<0.001	1.00	1.00–1.00	<0.001
Symptomatic	1.00	1.00–1.00	0.015	0.99	0.99–0.99	<0.001
Symptomatic with severe disease	0.99	0.99–0.99	<0.001	1.01	1.01–1.02	<0.001
Deaths	1.01	1.01–1.01	<0.001	0.99	0.98–0.99	0.009

Notes: * independent variables; IRR—incidence rate ratio; 95% CI—confidence interval; *p*—*p* value.

**Table 2:** Behavioral, lifestyle, and nutritional variables of the young older adults and the nonagenarians/centenarians.

**Model 3**	**Younger Older Adults (Outcome)** **Log Likelihood= −10343.7, x^2^ = 590.11 (7 df), *p* < 0.001**			**Nonagenarians/Centenarians (Outcome)** **Log Likelihood= −7480.3,** **x^2^ = 2687.25 (11 df), *p* < 0.001**		
Behavioral and Mental Health Variables *	**IRR**	**95% CI**	***p***	**IRR**	**95% CI**	***p***
Use of drugs	1.00	0.99–1.00	0.070	1.00	0.99–1.00	0.127
Tobacco	1.00	1.00–1.00	<0.001	0.99	0.99–1.00	<0.001
Alcohol	0.99	0.99–1.00	0.212	1.00	1.00–1.00	0.001
Alcohol addition	1.00	1.00–1.00	<0.001	1.06	1.05–1.07	<0.001
Smoking addition	1.00	1.00–1.00	0.780	0.99	0.99–0.99	<0.001
Psychosomatic problems	1.00	1.00–1.00	<0.001	0.99	0.99–0.99	<0.001
Depression	0.99	0.99–1.00	0.458	1.01	1.01–1.01	<0.001
Nonagenarians/centenarians grateful for a long life						
- Undecided				1 ^a^	1 ^a^	1 ^a^
- No				1.66	1.58–1.75	<0.001
- Yes				0.97	0.91–1.04	0.468
Nonagenarians/centenarians suffered the burden of not dying						
- Undecided	-	-	-	1 ^a^	1 ^a^	1 ^a^
- No				1.06	1.01–1.11	0.006
- Yes				0.57	0.53–0.61	<0.001
**Model 4**	**Importance of Nutrition in YOA (Outcome)** **Log Likelihood= −8822.6,** **x^2^ =2873.08 (15 df), *p* < 0.001**			**Importance of Nutrition in N/C (Outcome)** **Log Likelihood= −7728.0,** **x^2^ =3632.16 (16 df), *p* < 0.001**		
Nutritional Variables *	**OR**	**95% CI**	***p***	**OR**	**95% CI**	***p***
Variety and freshness of foods	0.08	0.06–0.12	<0.001	0.37	0.27–0.50	<0.001
Consumptions of ready to eat meals	3.06	2.18–4.28	<0.001	0.94	0.70–1.28	0.72
Bottled water	1.67	1.15–2.41	0.006	6.63	4.27–10.30	<0.001
Tap water	1.42	0.97–2.07	0.06	0.40	0.27–0.62	<0.001
Meat consumption	1.79	1.19 -2.68	0.004	0.94	0.63–1.38	0.75
Fish consumption	0.09	0.04–0.17	<0.001	1.44	0.90–2.31	0.12
Specific diets	0.62	0.31–1.21	0.16	1.00	1.00–1.01	0.03
Overweight	1.01	1.01–1.02	<0.001	1.01	1.00–1.02	0.008

Notes: * independent variables; IRR—incidence rate ratio; OR—odds ratios; 95% CI—confidence interval; *p*—*p* value; YOA—young older adults; N/C—nonagenarians/centenarians. ^a^—reference category.

**Table 3:** Social and community predictors of young older adults and nonagenarians/centenarians.

Model 5	Young Older Adults (Outcome) Log Likelihood= −6680.1, x^2^ =1059.75 (12 df), *p* < 0.001			Nonagenarians/Centenarians (Outcome) Log Likelihood= −6778.0, x^2^ =756.26 (11 df), *p* < 0.001		
Social and Community Gradient Variables *	IRR	95% CI	*p*	IRR	95% CI	*p*
Living with family member	1.00	0.99–1.00	0.610	0.99	0.99–0.99	<0.001
Living alone with family support	0.99	0.99–0.99	<0.001	1.00	0.99–1.00	0.160
Living alone with third-party support (neighbors, caregivers)	1.00	1.00–1.00	<0.001	0.99	0.99–0.99	<0.001
Living in nursing homes or shared apartments	1.00	0.99–1.00	0.971	1.00	1.00–1.00	<0.001
Continuing in professional positions	0.93	0.87–0.99	0.042	0.59	0.51–0.68	<0.001
Honorary achievement	0.93	0.91–0.95	<0.001	0.75	0.71–0.79	<0.001
Physical activities (gardening, yoga, sports, etc.)	1.48	1.42–1.54	<0.001	0.69	0.60–0.78	<0.001
Cultural activities	0.66	0.59–0.72	<0.001	0.59	0.53–0.66	<0.001
Social life	1.21	1.11–1.31	<0.001	0.32	0.27–0.40	<0.001
Parish activities	0.82	0.79–0.84	<0.001	1.28	1.20–1.36	<0.001
Bar meetings	1.04	1.02–1.07	<0.001	1.40	1.32–1.50	<0.001
Association activities	0.92	0.87–0.96	0.001	1.14	1.03–1.27	0.009

Notes: * independent variables; IRR—incidence rate ratio; 95% CI—confidence interval; *p*—*p* value

**Table 4:** Evaluation of the health care system in Salerno province by the male and female medical gender.

Model 6	Male and Female Physicians (Outcome)		
Structural Variables of Health *	OR	95% CI	*p*
Hospital equipment			
good	1 ^a^		
so-so	1.63	1.09–2.43	0.015
poor	2.49	1.50–4.14	<0.001
Cooperations between institutions			
good	1 ^a^		
so-so	0.25	0.13–0.47	<0.001
poor	0.05	0.02—0.11	<0.001
Public health information			
good	1 ^a^		
so-so	1.09	0.69–1.71	0.701
poor	1.04	0.60–1.79	0.888
Cooperation between clinics and primary care physicians			
good	1 ^a^		
so-so	5.45	2.93–10.16	<0.001
poor	25.76	12.32–53.84	<0.001
Cooperations between clinics and rehabilitation facilities			
good	1 ^a^		
so-so	0.58	0.34–0.97	0.041
poor	0.37	0.19–0.72	0.004
Cooperations between hospitals and specialty clinics			
good	1 ^a^		
so-so	0.44	0.25–0.77	0.004
poor	0.58	0.29–1.15	0.124
Self-help group			
good	1 ^a^		
so-so	1.21	0.64–2.26	0.544
poor	0.90	0.46–1.76	0.773

Notes: * independent variables; OR—odds ratios; 95% CI—confidence interval; *p*—p value. ^a^—reference category.

## Data Availability

The data included in this manuscript were provided by physicians of Salerno province. Therefore, we are not authorized to share the data with third party organizations. However, the corresponding author is available to provide any explanation to the Editor if requested.

## Supplementary Materials

The following supporting information can be downloaded at: https://www.mdpi.com/article/10.3390/nu14173665/s1, S1: Questionnaire on clinical status, nutritional behavior, and lifestyle, and social and community determinants of patient well-being from the perspective of physicians; S2: Graph on the medical field of physicians who responded to the questionnaire.
