# Supplementary material for: Clinical Status, Nutritional Behavior, and Lifestyle, and Determinants of Community Well-Being of Patients from the Perspective of Physicians: A Cross-Sectional Study of Young Older Adults, Nonagenarians, and Centenarians in Salerno and Province, Italy
Source: Nutrients. 2022 Sep 5;14(17):3665. doi: 10.3390/nu14173665 (PMC9459717; doi:10.3390/nu14173665)
Supplement: Supplementary file 1 [file nutrients-14-03665-s001.zip › S1-questionnaire old and centenarians from physicians perspec_en.pdf]

University of Salerno Department of Medicine, Surgery and Dentistry "Scuola Medica Salernitana" Section of Hygiene and Preventive Medicine

Dear Participant,

we are conducting this survey to better understand health or illness conditions, nutritional behavior, lifestyle, and determinants of community well-being in old age from the perspective of physicians, particularly geriatricians, gerontologists, and physicians working in nursing homes or rehabilitation facilities. The observations of primary care physicians working in the community are also of great importance.

By participating in this survey, you will be making an important contribution to health research in Italy, which is why we are asking you to participate.

I ask you to fill out the questionnaire as soon as possible. If you need to pause your response, you may continue at any time. If so, please click on the link to the online questionnaire in the invitation email.

For any questions please contact Dr. Silvana Mirella Aliberti (sialiberti@unisa.it).

A1. **What percentage of patients you have treated in the last three years were old or centenarians? Please estimate the percentage by moving the slider.**

Old people ☐

Centenarians ☐

A2. **Please estimate the percentage of older people who...**

on the whole healthy ☐

despite age-related illnesses/chronic diseases, their abilities are not very limited overall ☐

clearly marked by the disease ☐

severe suffering ☐

A3. **Please estimate the percentage of centenarians who...**

on the whole healthy ☐

despite age-related illnesses/chronic diseases, their abilities are not very limited overall ☐

clearly marked by the disease

severe suffering

A4. Which diseases occur most frequently in older people? Please sort the diseases according to the frequency of their occurrence.

Cardiovascular diseases (high blood pressure, stroke, heart attack, etc.)

Respiratory diseases (sinusitis, chronic bronchitis, etc.)

Blood diseases (anemia, leukemia, etc.)

Rheumatic diseases (athritis etc.)

Metabolic diseases (diabetes, thyroid diseases, etc.)

Gastrointestinal diseases (gastritis, colorectal, gastric and pancreatic tumors, etc.)

Neurological diseases (dementia, Parkinson's disease, multiple sclerosis, etc.)

Liver diseases (hepatits etc.)

Cancer

Other diseases

A5. Which diseases occur most frequently in centenarians? Please sort the diseases according to the frequency of their occurrence.

Cardiovascular diseases (high blood pressure, stroke, heart attack, etc.)

Respiratory diseases (sinusitis, chronic bronchitis, etc.)

Blood diseases (anemia, leukemia, etc.)

Rheumatic diseases (athritis etc.)

Metabolic diseases (diabetes, thyroid diseases, etc.)

Gastrointestinal diseases (gastritis, colorectal, gastric and pancreatic tumors, etc.)

Neurological diseases (dementia, Parkinson's disease, multiple sclerosis, etc.)

Liver diseases (hepatits etc.)

Cancer

Other diseases

A6. What do you estimate the percentage of comorbidities in the old?

Comorbidities older people

A7. **What do you estimate the percentage of comorbidities in the centenarians?**

Comorbidities centenarians ☐☐☐☐☐☐☐☐☐☐

B1. **Can you estimate how many drugs the average old and centenarians take?**

Old people ☐☐☐☐☐☐☐☐☐☐

Centenarians ☐☐☐☐☐☐☐☐☐☐

B2. **Please estimate what percentage of the old and centenarians use tobacco regularly?**

Old people ☐☐☐☐☐☐☐☐☐☐

Centenarians ☐☐☐☐☐☐☐☐☐☐

B3. **Please estimate what percentage of the old and centenarians regularly consume alcohol?**

Old people ☐☐☐☐☐☐☐☐☐☐

Centenarians ☐☐☐☐☐☐☐☐☐☐

B4. **Please estimate what percentage of the old and centenarians suffer from alcohol dependence?**

Old people ☐☐☐☐☐☐☐☐☐☐

Centenarians ☐☐☐☐☐☐☐☐☐☐

B5. **Please estimate what percentage of smokers among the old and centenarians exhibit addictive behaviors?**

Old people ☐☐☐☐☐☐☐☐☐☐

Centenarians ☐☐☐☐☐☐☐☐☐☐

C1. **How high do you rate the importance of nutrition in the care of your patients?**

	very high	rather high	neither	rather low	very low	do not know
Nutrition importance	☐	☐	☐	☐	☐	☐

C2. **How important are the following aspects for the nutrition of older people:**

	very important	important	rather important	rather unimportan t	unimportan t	totally unimportan t	do not know
Variety and freshness of (often regional) foods	☐	☐	☐	☐	☐	☐	☐
Consumption of ready meals	☐	☐	☐	☐	☐	☐	☐
Daily consumption of bottled water	☐	☐	☐	☐	☐	☐	☐

	very important	important	rather important	rather unimportan t	unimportan t	totally unimportan t	do not know
Daily consumption of tap water	☐	☐	☐	☐	☐	☐	☐
Share of meat consumption in the diet	☐	☐	☐	☐	☐	☐	☐
Proportion of fish consumption in the diet	☐	☐	☐	☐	☐	☐	☐
Specific diets against diseases and obesity	☐	☐	☐	☐	☐	☐	☐

C3. How important are the following aspects for the nutrition of centenarians?

	very important	important	rather important	rather unimportan t	unimportan t	totally unimportan t	do not know
Variety and freshness of (often regional) foods	☐	☐	☐	☐	☐	☐	☐
Consumption of ready meals	☐	☐	☐	☐	☐	☐	☐
Daily consumption of bottled water	☐	☐	☐	☐	☐	☐	☐
Daily consumption of tap water	☐	☐	☐	☐	☐	☐	☐
Share of meat consumption in the diet	☐	☐	☐	☐	☐	☐	☐
Proportion of fish consumption in the diet	☐	☐	☐	☐	☐	☐	☐
Specific diets against diseases and obesity	☐	☐	☐	☐	☐	☐	☐

C4. Estimating the proportion of older people and centenarians who are overweight.

Old people ____________

Centenarians ____________

D1. What is the estimated percentage of old and centenarians who suffer from psychosomatic health problems (symptoms such as insomnia, constipation, weight loss, fatigue, etc.)?

Old people ____________

Centenarians ____________

D2. What is the estimated percentage of old and centenarians who suffer from depression?

Old people ____________

Centenarians ____________

E1. **What percentage of older people and centenarians are on their own or receive constant support?Life in the family**

Old people ☐☐☐☐☐☐☐☐☐☐

Centenarians ☐☐☐☐☐☐☐☐☐☐

E2. **What percentage of older people and centenarians are on their own or receive constant support?Living alone with family support**

Old people ☐☐☐☐☐☐☐☐☐☐

Centenarians ☐☐☐☐☐☐☐☐☐☐

E3. **What percentage of older people and centenarians are on their own or receive constant support?Living alone with support from third parties (neighbors, care services, etc.)**

Old people ☐☐☐☐☐☐☐☐☐☐

Centenarians ☐☐☐☐☐☐☐☐☐☐

E4. **What percentage of older people and centenarians are on their own or receive constant support?Living in senior and nursing facilities or shared apartments**

Old people ☐☐☐☐☐☐☐☐☐☐

Centenarians ☐☐☐☐☐☐☐☐☐☐

F1. **How important do you consider the following aspects of lifestyle to be for a fulfilling old age?**

	very important	important	rather important	rather unimportan t	unimportan t	totally unimportan t	do not know
Continuation of professional tasks	☐	☐	☐	☐	☐	☐	☐
Honorary office (Ital.: carica onorifica)	☐	☐	☐	☐	☐	☐	☐
Physical activities (gardening, sports, yoga, etc.)	☐	☐	☐	☐	☐	☐	☐
Fixed rituals (ital.: rituali fissati)	☐	☐	☐	☐	☐	☐	☐
Social contacts	☐	☐	☐	☐	☐	☐	☐
Parish	☐	☐	☐	☐	☐	☐	☐
Coffee shop/regular coffee meetings	☐	☐	☐	☐	☐	☐	☐
Associations	☐	☐	☐	☐	☐	☐	☐

G1. **Are the majority of centenarians especially grateful for a long life?**

- Yes ☐
- No ☐
- undecided ☐

G2. **Do the majority of centenarians suffer particularly often from the "burden of not being able to die"?**

- Yes ☐
- No ☐
- undecided ☐

H1. **From your professional experience, how do you evaluate the health care system in the province of Salerno?**

	very good	good	partly partly	poor	Very poor	not specified
Hospital Equipment	☐	☐	☐	☐	☐	☐
Cooperation of the main institutions	☐	☐	☐	☐	☐	☐
Informing the public about health care	☐	☐	☐	☐	☐	☐
Cooperation between clinics and physicians in private practice	☐	☐	☐	☐	☐	☐
Cooperation between clinics and rehab facilities	☐	☐	☐	☐	☐	☐
Cooperation between clinics and specialist clinics	☐	☐	☐	☐	☐	☐
Self-help groups	☐	☐	☐	☐	☐	☐

I1. **How many of the old and centenarians you treat have tested positive for SARS-CoV-2 virus?**

- Asymptomatic ☐☐☐☐☐☐☐☐☐☐
- Paucisintomatic ☐☐☐☐☐☐☐☐☐☐
- Symptomatic ☐☐☐☐☐☐☐☐☐☐
- Symptomatic with severe disease ☐☐☐☐☐☐☐☐☐☐
- Deaths ☐☐☐☐☐☐☐☐☐☐

J1. **Your gender?**

- Male ☐
- Female ☐
- Other ☐

Other

J2. **How old are you?**

J3. **Education and practice of the specialist (physician)?**

- General Medicine ☐
- Gerontology ☐
- Surgery ☐
- Internal medicine ☐
- Cardiology ☐
- Genetics ☐
- Gastroenterology ☐
- Gynecology ☐
- Nephrology ☐
- Psychology ☐
- Orthopedics ☐
- Dentistry ☐
- Ophthalmology ☐
- Hematology ☐
- Diabetology ☐
- Endocrinology ☐
- Sports Medicine ☐

Other

Other

Thank you for your participation!
