# Supplementary material for: Clinical Status, Nutritional Behavior, and Lifestyle, and Determinants of Community Well-Being of Patients from the Perspective of Physicians: A Cross-Sectional Study of Young Older Adults, Nonagenarians, and Centenarians in Salerno and Province, Italy
Source: Nutrients. 2022 Sep 5;14(17):3665. doi: 10.3390/nu14173665 (PMC9459717; doi:10.3390/nu14173665)

Figure S2. Medical field of physicians who responded to the questionnaire. Number for each item may not add up to total number of study population due to missing value.

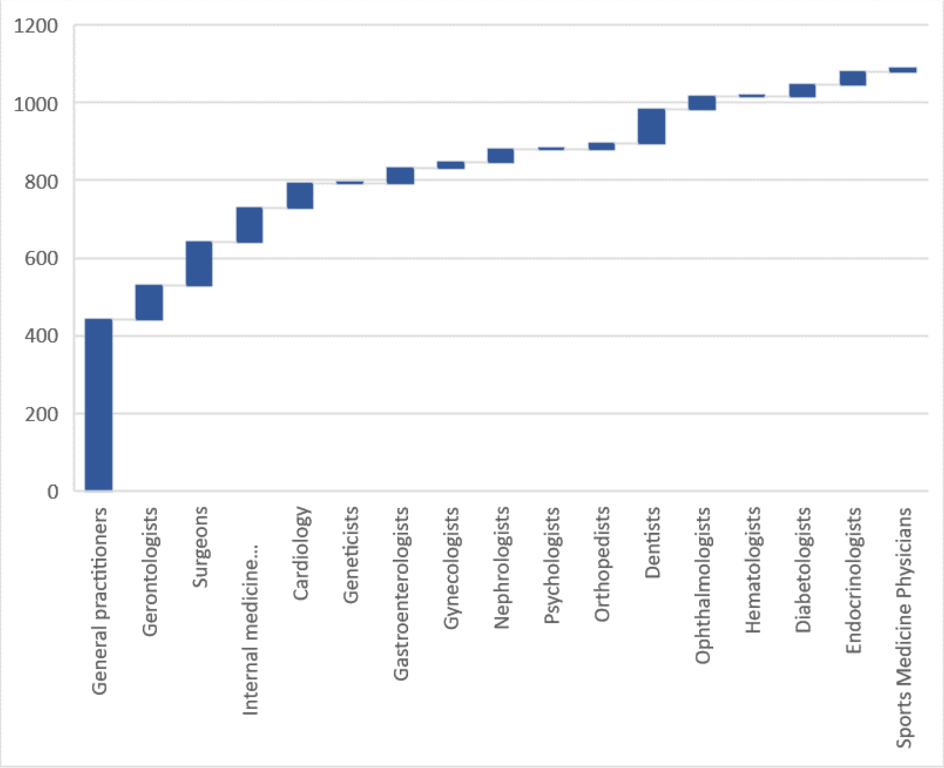

Supplement: Supplementary file 1 [file nutrients-14-03665-s001.zip › Supplementary material S2.pdf]
